# Commentary: Tailored therapy for *Helicobacter pylori* eradication: A systematic review and meta-analysis

**DOI:** 10.3389/fphar.2022.1090776

**Published:** 2022-12-20

**Authors:** Toshihiko Kakiuchi, Masato Yoshiura

**Affiliations:** Department of Pediatrics, Faculty of Medicine, Saga University, Saga, Japan

**Keywords:** *Helicobacter pylori*, microbial sensitivity tests, clarithromycin, eradication therapy, antibiotics, drug resistance, esophagogastroduodenoscopy, treatment duration

## 1 Introduction

Ma et al. (2022) published “Tailored therapy for *Helicobacter pylori* eradication,” a systematic review and meta-analysis suggesting that a tailored therapeutic approach might provide a better eradication success rate than empirical therapeutic methods in the first-line treatment regimen for *H. pylori*. To be considered a first-line treatment for *H. pylori*, a regimen must have an eradication rate of at least 80%–85% (Fallone et al., 2016). Therefore, various guidelines for the treatment of *H. pylori* recommend antibiotic susceptibility testing prior to therapy initiation to maintain high eradication rates (Jones et al., 2017; Kato et al., 2019; Kato et al., 2020; Jung et al., 2021; Romano et al., 2022). In recent guidelines for the treatment of *H. pylori*, non-bismuth quadruple therapy, sequential or concomitant treatment, and bismuth quadruple therapy is recommended as first-line treatment regimens in areas where clarithromycin (CAM) resistance mutations cause >15% of all cases (Malfertheiner et al., 2017; Liu et al., 2018). However, there are concerns that the inappropriate use of antibiotics may eventually lead to drug resistance. Because the overall *H. pylori* antibiotic resistance rates for CAM are 17.2% (95%, confidence interval 16.5%–17.9%) (De Francesco et al., 2010), it is necessary to consider performing a CAM resistance test before eradication therapy for *H. pylori*.

## 2 Antibiotic susceptibility testing for *H. pylori*

With increasing CAM resistance worldwide, CAM resistance tests are becoming vital. However, there is concern regarding antibiotic susceptibility testing, which is the basis of tailored therapy for *H. pylori* as shown by Ma et al. (2022).

### 2.1 Culturing for drug susceptibility testing for *H. pylori*

Culturing for *H. pylori* drug susceptibility testing is seldom implemented due to the current lack of testing method standardization or consensus on the antibiotic resistance breakpoints (Li et al., 2022). In Japan, culturing for *H. pylori* drug susceptibility testing is not covered by public health insurance in some areas. In children, esophagogastroduodenoscopy (EGD) to collect specimens is highly invasive and may be technically limited to facilities where it can be performed. Additionally, in Japan, screening for *H. pylori* is being expanded to include junior and senior high school students (Kakiuchi et al., 2019) and targets asymptomatic students to detect infection and prevent gastric cancer by non-invasive methods, including urine, blood, and stool analyses. Because the screening is carried out without EGD, performing antibiotic susceptibility testing for *H. pylori* using culture is difficult.

### 2.2 Molecular detection to measure drug susceptibility for *H. pylori*

The molecular method of drug susceptibility testing for *H. pylori* uses real-time polymerase chain reaction (PCR) and fluorescent *in situ* hybridization on stool and stomach samples. Attempts to test for *H. pylori* CAM resistance using non-invasive or invasive methods have been reported (Xiong et al., 2013). However, these methods have been difficult to implement in clinical settings because they require individual clinicians to use a machine that is large and high-priced, which limits the number of medical establishments where it can be set up. Additionally, the method requires technical skill.

## 3 Discussion

The possibility reported by Ma et al. (2022)—tailored therapy that might provide a better eradication rate than empirical methods in the first-line treatment regimen for *H. pylori*—is important because it would inhibit bacterial growth through the appropriate use of antibacterial drugs. However, implementation in clinical settings would be difficult.

We developed a new reagent for evaluating *H. pylori* genes and CAM resistance mutations, using stool samples, developed as a dedicated reagent for the Smart Gene™ point-of-care testing kit (Kakiuchi et al., 2020; Kakiuchi et al., 2022). Smart Gene™ (Mizuho Medy Co., Ltd., Tosu-City, Saga, Japan) was invented in accordance with the idea of point-of-care testing, and it can mechanically do nucleic acid extraction and amplification and detection of the target genes. It is compact, light, and cheap gene detection equipment ($4,500 per unit). The reagent used in Smart Gene™ for *H. pylori* is also inexpensive, costing around $25 per sample. To the best of our knowledge, this is the world’s first reagent for point-of-care testing capable of rapidly detecting *H. pylori* genes and CAM resistance mutations. The assay is based on PCR and the quenching-probe technique, which detects *H. pylori* genes and CAM resistance mutations concurrently in about 50 min per sample. The measurement time is far briefer than those of conventional methods.

Similarly, Tsuda et al. developed a kit that can detect *H. pylori* genes and CAM resistance mutations in gastric fluid samples using Smart Gene^TM^ within 60 min (Tsuda et al., 2022). The test kit, combined with EGD, can be beneficial for the determination of CAM resistance mutations when it is difficult to test for these by culturing. Another major advantage of this method is that results can be obtained faster than with culture methods.

## 4 Conclusion

In order to expand tailored therapy for *H. pylori* eradication, it is essential to further develop and expand simple and economical methods that can test the antibiotic susceptibility of *H. pylori*.
